# Inborn Errors of Immunity and Cancer

**DOI:** 10.3390/biology10040313

**Published:** 2021-04-09

**Authors:** Alessandra Tiri, Riccardo Masetti, Francesca Conti, Anna Tignanelli, Elena Turrini, Patrizia Bertolini, Susanna Esposito, Andrea Pession

**Affiliations:** 1 Pediatric Clinic, Pietro Barilla Children’s Hospital, University of Parma, 43126 Parma, Italy; atiri@ao.pr.it (A.T.); annatignanelli@gmail.com (A.T.); elena.turrini3@gmail.com (E.T.); 2 Pediatric Unit, IRCCS Azienda Ospedaliero-Universitaria di Bologna, University of Bologna, 40138 Bologna, Italy; riccardo.masetti5@unibo.it (R.M.); francescac.ageop@aosp.bo.it (F.C.); andrea.pession@unibo.it (A.P.); 3 Pediatric Oncohematology Unit, Pietro Barilla Children’s Hospital, 43126 Parma, Italy; pbertolini@ao.pr.it

**Keywords:** cancer, inborn errors of immunity, children

## Abstract

**Simple Summary:**

Inborn Errors of Immunity (IEI) are a heterogeneous group of disorders characterized by a defect in the function of at least one, and often more, components of the immune system. The overall risk for cancer in children with IEI ranges from 4 to 25%. Several factors, namely, age of the patient, viral infection status and IEI type can influence the development of different cancer types. Immunologists and oncologists should interact to monitor and promptly diagnose the potential development of cancer in known IEI patients, as well as an underlying IEI in newly diagnosed cancers with suggestive medical history or high rate of therapy-related toxicity. The creation of an international registry of IEI cases with detailed information on the occurrence of cancer is fundamental to optimizing the diagnostic process and to evaluating the outcomes of new therapeutic options, with the aim of improving prognosis and reducing comorbidities.

**Abstract:**

Inborn Errors of Immunity (IEI) are a heterogeneous group of disorders characterized by a defect in the function of at least one, and often more, components of the immune system. The aim of this narrative review is to discuss the epidemiology, the pathogenesis and the correct management of tumours in patients with IEI. PubMed was used to search for all of the studies published over the last 20 years using the keywords: “inborn errors of immunity” or “primary immunodeficiency” and “cancer” or “tumour” or “malignancy”. Literature analysis showed that the overall risk for cancer in children with IEI ranges from 4 to 25%. Several factors, namely, age of the patient, viral infection status and IEI type can influence the development of different cancer types. The knowledge of a specific tumour risk in the presence of IEI highlights the importance of a synergistic effort by immunologists and oncologists in tracking down the potential development of cancer in known IEI patients, as well as an underlying IEI in patients with newly diagnosed cancers. In the current genomic era, the creation of an international registry of IEI cases integrated with malignancies occurrence information is fundamental to optimizing the diagnostic process and to evaluating the outcomes of new therapeutic options, with the hope to obtain a better prognosis for these patients.

## 1. Introduction

Inborn Errors of Immunity (IEI) are a heterogeneous group of disorders characterized by a defect in the function of at least one, and often more, components of the immune system. These defects lead to increased susceptibility to infections, immune dysregulation phenotypes (e.g., severe atopy, autoimmunity, polyclonal lymphoproliferation) and a predisposition towards the onset of malignancies [1,2,3,4,5,6,7]. Over 180 defined IEI and new clinical entities are being recognized thanks to new molecular tests, which have increased the number of genes related to IEI to over 430 [8].

The incidence of all currently recognized paediatric IEI taken together is approximately 1:2000 for children [9], although severe forms such as severe combined immune deficiency (SCID) are extremely rare (approximately, 1:70,000) [10,11]. The early detection of patients with IEI is critically important, as effective therapy is available for most of the different disorders but is most beneficial when instituted before target organ damage has occurred by infection or immune dysregulation phenotypes [12,13]. Similarly, early recognition of IEI may lead to a precise genetic diagnosis, which in turn may be of great importance for prenatal genetic counselling, prediction of specific potential disease’s complications and eventual targeted therapy [14]. Although the time taken to diagnose IEI has decreased in recent years, the average delay in diagnosis is 1.9 years for adults and children, resulting in significant morbidity and mortality [12].

IEI are divided into two main groups: deficiencies of innate immunity and deficiencies of adaptive immunity. Defects in innate immunity comprise phagocytes disorders, Toll-like receptor (TLR)-mediated signalling and complement [15]. Defects in adaptive immune responses include antibody deficiency syndromes, combined immunodeficiency (CID) and SCID [11,16,17,18]. The earliest evidence that individuals with IEI develop cancer was reported in 1963 [19,20]. In recent decades, advances in the prevention and treatment of infections, paired with the accessibility and convenience of immunoglobulin (Ig) replacement therapy, have led to increased overall survival and prolonged life expectancy of patients affected by IEI. Patients’ increased longevity has been accompanied by the emergence of cancer as the second-leading cause of death in IEI after infections [21,22,23]. This narrative review aims to discuss the epidemiology, the pathogenesis and the correct management of tumours in patients with IEI, thus highlighting the importance of an early diagnosis and personalized therapeutic strategies. PubMed was used to search for all of the studies published over the last 20 years using the keywords: “primary immunodeficiency” and ‘Inborn Errors of Immunity’ and “cancer” or “tumour” or “malignancy”. More than 2500 articles were found, but only those published in English or providing evidence-based data were included in the evaluation. Table 1 summarizes the abbreviations used in this manuscript.

## 2. Epidemiology of the Association between IEI and Cancer

The overall risk for cancer in children with IEI ranges from 4 to 25% [21,24,25], with different rates among the various diseases. The exact incidence rates for malignancies occurring in the presence of IEI are difficult to ascertain, given the rarity of IEI [26] and the possibility that in many cancer patients, the underlying IEI is not diagnosed, especially when it presents as a mild or atypical clinical manifestation or with a late onset, which leads to underestimation [27]. One analysis of case reports provided a risk of 0.7–15% [28]. A recently published series of studies, which included large cohorts and a comparison group from Australia [29], The Netherlands [30], and the USA [31], indicated that patients with IEI have a significantly increased risk of all cancers of 1.6-, 2.3-, or 1.42-fold, respectively, compared to the general population, much lower than the approximately 10,000-fold increase estimated in past reports.

In the early 1970s, the Immunodeficiency Cancer Registry (ICR) was established by the University of Minnesota. Despite limitations due to voluntary submission of data and changes in diagnosis methods and malignancies’ classifications, the ICR database remains one of the most authoritative epidemiological analysis tool [22,32]. According to the ICR database, non-Hodgkin’s lymphoma (NHL) and Hodgkin’s disease (HD) account for 48.6% and 10%, respectively, of the malignancies seen in patients with IEI [22]. Malignancy occurs only in patients with a few subtypes of IEI [33]. More than half of IEI-related cancer cases have been reported in patients with ataxia–telangiectasia (A–T) and common variable immunodeficiency (CVID). One-third of the cases are associated with Wiskott–Aldrich syndrome (WAS), SCID and selective IgA deficiency [33]. The increased frequency of bone marrow transplantation in patients affected by SCID and WAS ensures the complete reconstitution of the immune system, which could justify the reduction in the frequency of malignancy observed in the last years. In most IEI associated with cancer, B cell function is at least partially defective, whereas T cell function ranges from normal to impaired [23,34,35]. 

A recent retrospective study reported the incidence of tumours among 690 patients with a diagnosis of IEI made between 1990 and 2017 in Brescia, Italy [36]. The overall incidence was 3.6%, with discrepancies among the different IEI considered. Haematological neoplasms were more frequent than solid tumours, with NHL being the most frequent. These lymphomas were frequently extranodal-onset and showed a B cell phenotype prevalence, particularly, diffuse large B cells [36]. The average age at onset was 20 years [37]. In CVID, which usually manifests in young adulthood, malignancy also had a late onset. In patients with SCID and CID, the peak of onset was described between 0 and 10 years [38], and mortality was high without bone marrow transplantation.

The latency between the diagnosis of IEI and the first tumour’s onset varies from case to case. Malignancy can also be the first clinical expression of the underlying immunodeficiency, particularly in those IEI patients that present a delayed onset or a mild clinical manifestation [34]. Therefore, screening investigations for the most frequent tumours should be performed in patients with IEI.

## 3. Pathogenesis of Cancer in IEI

The mechanisms that explain the increased susceptibility of IEI patients to the development of tumours are multiple and different in the various pathologies. The most common process is the reduction of cell-mediated immunosurveillance (as occurs in combined immunodeficiency), which plays a fundamental role in protecting against tumours [39,40,41,42]. Several pathways, such as genomic instability, overstimulation of immune cells, viral infection and chronic inflammation, have been proposed to explain the increased incidence of malignancy among patients with IEI [43]. Moreover, defective dendritic cells (DCs) differentiation and function, which affect the initiation and development of T cell responses, have been associated with cancer development [44].

### 3.1. Genomic Instability

DNA instability leads to an enhanced risk of cancer. This condition occurs in the following cases: (1) defects in the DNA damage response (i.e., A–T); (2) defects in apoptosis that, causing cellular immortalization, allow cells to survive even in the presence of irreversible damage to the genome (i.e., autoimmune lympho-proliferative syndrome); (3) deficiencies in the checkpoints of the cell cycle, which is fundamental for correct repair of damage (i.e., cartilage–hair hypoplasia); (4) defects in cytokinesis, which, by hindering cell division, lead to the formation of genetically unstable tetraploid cells (i.e., neutropenia X-linked and WAS) [45,46,47,48].

The DNA damage response is normally responsible for sensing and repairing damaged DNA [49] and is one of the most powerful tumour surveillance mechanisms [50]. Lymphocytes have intrinsic mechanisms that are part of the immune diversity generation process, such as V(D)J recombination, class switch recombination (CSR) and somatic hypermutation (SMH) [45]. In the case of a deficient DNA repair system, the DNA is continually exposed to endogenous mutagen metabolites and external factors such as ultraviolet rays, ionizing radiation and chemicals, which necessarily cause the appearance of mutations that favour cell degeneration towards neoplastic forms [51,52]. In defects in apoptosis, the acquisition of mutations is favoured by the presence of apoptosis deficiency, preventing the cell from meeting a programmed natural death following the appearance of DNA damage [46]. In some IEI patients, the acquisition of mutations is favoured by the presence of defects in cell cycle checkpoints, which normally detect DNA damage and stop the cell cycle for the time necessary to guarantee a correct repair [46]. Instead, cell cycle checkpoints are absent in these pathologies. Finally, alterations in cytokinesis determine the formation of genetically unstable tetraploid cells, which can then transform into aneuploid tumour cells [11].

### 3.2. Viral Infections

Infection plays a decisive role in the genesis of haematologic and solid tumours through activation of oncogenic viruses and chronic antigenic stimulation. Some estimates suggest that >20% of carcinomas in patients with IEI are infection-induced [34]. It seems that 12% of cancers in humans are related to viral infections, and the percentage is even higher in patients with IEI [53,54], in whom the incidence of cancer involving organs that are targeted by viral infection is increased [42].

The inability to eliminate viral pathogens creates a hostile inflammatory environment that promotes cell survival and proliferation [54,55,56,57,58,59,60]. Immune dysregulation leads to reduced clearance of viruses, such as Epstein–Barr virus (EBV), hepatitis B virus, hepatitis C virus, human papilloma virus (HPV), human herpes virus-6 (HHV-6), HHV-8, human T cell lymphotropic virus and Kaposi sarcoma-associated virus, which contribute to cellular immortalization and transformation and collectively account for 10 to 15% of cancers worldwide [53,61]. 

Among oncogenic viruses, EBV is the most frequently associated with malignancy in IEI patients because of its high prevalence (95%) and ability to transform epithelial cells, B cells, T cells and NK cells [62,63]. According to the ICR data, EBV is found in 30–60% of patients with lymphoproliferative disorders and IEI [29]. The role of EBV has been hypothesized for many different B cell lymphoproliferative disorders (BLPDs) in patients with IEI, including virus-associated haemophagocytic syndrome, Burkitt’s lymphoma, classic Hodgkin’s lymphoma, posttransplant lymphoproliferative disorders (PTLDs) and HIV-associated lymphoproliferative disorders (LPDs) [64].

EBV has a particular affinity for B cells, which act as a reservoir for the virus. Thus, B cell lymphomas are the predominant malignancy associated with EBV infection [5]. EBV triggers the proliferation of B cells producing EBV-specific and EBV-nonspecific antibodies, followed by a cellular immune response involving cytotoxic T lymphocytes (CTLs). The absence of an appropriate T cell response can lead to EBV-driven B cell proliferation [22,65]. Furthermore, EBV expresses genes that inhibit cell-mediated immunity, making it immortal within B cells, which proliferate uncontrollably under the viral stimulus, acquiring mutations due to loss of heterozygosity and/or cytogenetic rearrangements [46]. Based on genetic differences in EBV nuclear antigen 2 (EBNA-2), EBNA-3a and EBNA-3c latent genes, EBV has been classified into two major strains, which are referred to as EBV type 1 (EBV-1) and type 2 (EBV-2). While EBV-1 directly transforms B cells, EBV-2 infects T cells and is poorly transforming. Infection of T cells by EBV- 2 stimulates an inflammatory background, leading to oncogenesis in T cells [66].

Most IEI associated with susceptibility to EBV involve defects in T and/or NK cells, affecting CTL and NK cell cytotoxicity, which plays a major role in defence against viral pathogens [7,67]. These defects involve various steps in T and NK cell development and activation, ranging from impaired T cell receptor (TCR) gene rearrangement and thymic egress (e.g., Omenn syndrome, coronin1a deficiency, X-linked lymphoproliferative syndrome (XLP) type 1, interleukin-2-inducible T-cell kinase (ITK) deficiency, warts, hypogammaglobulinemia, infections, and myelokathexis (WHIM) syndrome, A–T and MST1 mutation) to poor long-term maintenance of cellular immunity and reduced cell activation (i.e., CD27 deficiency, XLP type 1, PI3K activation, DiGeorge Syndrome [DGS], WHIM syndrome, X-linked immunodeficiency with magnesium defect, Epstein-Barr virus infection, and neoplasia [XMEN] disease and ITK deficiency). Patients affected by IEI characterized by overproliferation of immortal B cells, such as that caused by activated PI3K, are also at risk of EBV-associated LPDs [62]. Conversely, defects in innate immunity are not associated with an increased risk for EBV-LPDs, which may be due to the less critical role of innate immunity in protecting against EBV complications [62].

Failure of both innate (NK cells) and adaptive (T cells) immune cellular mechanisms facilitates viral escape and subsequent viral transformation of lymphocytes with poor cytotoxic clearance of virally infected cells [68,69]. A recent review described IEI associated with EBV-induced LPDs. The authors found that a minority of patients, particularly those affected by CD27 deficiency, Di George syndrome, WHIM syndrome or autoimmune lymphoproliferative syndrome (ALPS), develop T cell LPDs. Improper naïve T cell expansion and T cell differentiation are thought to cause insufficient defence against the development of EBV-associated T cell LPDs [62]. Approximately one-third of the reported EBV+ B cell LPDs are Hodgkin lymphomas.

### 3.3. Chronic Antigen Stimulation

In IEI, the lack of T and B cell responses to antigens, which is normally followed by antigens elimination in an immunocompetent host, leads to antigen persistence in the body (pathogen-infected and/or transformed cells). By establishing a persistent inflammatory state, chronic infections create tissue damage, which can be the precursor of a subsequent malignant transformation, as seen in *Helicobacter pylori* infection which is implicated in gastric carcinoma and gastric mucosa-associated lymphoid tissue (MALT) lymphoma development [34,46]. The chronic presence of this bacterium causes the production of cytokines that promote ectopic proliferation of lymphoid tissue [46]. Sustained antigenic stimulation can lead to the progressive loss of cytokine secretion (interleukin [IL]-2 and tumour necrosis factor [TNF]-α), impairment of interferon (IFN)-γ production, and premature apoptosis of CD8+ T cells [70]. This quantitative and qualitative loss of the CD8+ T cell immune responses against tumour cells can eventually result in the development of malignancy, being specifically recognized by T lymphocytes.

## 4. Malignancy Patterns of Various IEI Subtypes

The type of malignancy that is seen is highly dependent on the specific IEI, the age of the patient, and probably the viral infection status, indicating that different pathogenic mechanisms may be implicated in each case (Table 2). Associations most frequently observed between IEI and tumours are reported below. 

### 4.1. Selective IgA Deficiency

Selective IgA deficiency is defined by the European Society of Immunodeficiency as a serum IgA level < 7 mg/dL with normal levels of serum IgG and IgM in an individual older than 4 years in whom other causes of hypogammaglobulinemia have been excluded and who has a normal IgG antibody response to vaccination [70]. It is the most prevalent type of immunodeficiency, with an estimated prevalence of ~1:600 [3]. 

IgA deficiency in children can occur without symptoms. However, considering that IgA deficiency and CVID share a common genetic basis, the progression from mild or asymptomatic selective IgA deficiency to CVID is possible [71]. Therefore, the clinical presentation is variable, ranging from an asymptomatic condition to recurrent infections, allergic diseases, autoimmune diseases, lymphoid and gastrointestinal malignancies. Recurrent infections are frequent in the cluster of symptomatic patients, because IgA deficiency leads to a defective mucosal defence against pathogens, especially of the respiratory and gastrointestinal tract. The second most common clinical manifestation is allergic disease, including asthma, atopic dermatitis, allergic rhinitis, urticaria and food allergies [72,73]. Regarding autoimmune diseases, there is a strong association between celiac disease (CD), thyroiditis and IgA deficiency, meaning that 2–3% of celiac patients have IgA deficiency, and approximately 8% of individuals with IgA deficiency have CD [74].

There are few and controversial studies about IgA deficiency with malignancies. The types of malignancies reported in patients with selective IgA deficiency are similar to those reported for CVID, especially lymphoid and gastric malignancies [75]. The increased prevalence of gastric cancer in patients with selective IgA deficiency could result from a diminished defence against *H. pylori* in the gastric mucosa, highlighting the immunoprotective role of IgA against this type of malignancy [76,77]. In a case report, a patient with selective IgA deficiency was described with γ/δ-type abdominal T cell NHL [78]. More recently, a case of primary cutaneous marginal zone lymphoma with sequential development of nodal marginal zone lymphoma has been reported in a patient with selective IgA deficiency [79]. A combined Danish and Swedish study of 562 patients with IgA deficiency and CVID did not show an increased risk for cancer in patients with IgA deficiency, while cancer risk was moderately elevated in the CVID cohort [26].

### 4.2. Common Variable Immune Deficiency (CVID)

CVID is the most common clinically significant IEI disorder characterized by hypogammaglobulinemia due to impaired B cell differentiation resulting in greater susceptibility to bacterial infections and malignancies [80], particularly lymphoma and gastric cancer. CVID affects both children and adults, with an estimated prevalence of 1:25,000–50,000 [81]. The number of B cells may be normal or reduced, while the serum levels of IgG are reduced to more than two standard deviations below the mean. Most patients also have decreased levels of IgA, and many have low IgM levels [75]. The pathogenesis of CVID is partially understood. Rare monogenic causes have been identified; in some cases, the identified genes are often linked to B cell dysfunction, while in other cases, the connection to antibody deficiency is less clear [82]. Several immunological studies of large cohorts of CVID patients have demonstrated phenotypic and functional abnormalities of B cells [83], T cells [84] and antigen-presenting cells [85]. These abnormalities include mutations that occur in genes essential for the cooperation between B and T cells in the germinal centre, as well as for intrinsic signalling pathways of such cells. 

Genetic mutations have been identified in genes involved in some aspects of B cell biology, including mutations in ICOS [86], CD19 [87], and TNF receptor superfamily members 13B (TNFRSF13B or TACI) [88,89] and 13C (TNFRSF13C or BAFF-R) [90]. In CVID, most patients suffer mainly from infections, while others are particularly prone to non-infectious complications. Non-infectious complications include recurrent sinopulmonary infections, autoimmune disorders, malabsorptive symptoms and malignancies [91].

Generally, malignancy in CVID patients usually occurs during the 4th–6th decade of life. The gastrointestinal tract [92] and lymphoid tissue are among the most affected systems [93], as shown by the fact that CVID patients have an almost 47-fold increased risk for gastric cancer and a 30-fold increased risk for lymphoma compared to the general population [94]. In a recent meta-analysis [95] that included 48 studies with a total of 8123 CVID patients, the overall prevalence of malignancy was 8.6%.

Lymphoma is the most frequent malignancy, followed by epithelial tumours of the stomach, thymus, breast, bladder and cervix. Lymphoma is one of the more severe complications of CVID, and its development seems to be multifactorial with the interplay of genetics, immune dysregulation and chronic infectious agents, including non-oncogenic and oncogenic viruses such as EBV [96]. CVID-associated lymphomas are rarely seen in children and usually occur in the fourth to seventh decades of life. They are most frequently B cell NHLs. NHL is often extranodal and usually EBV-negative and can arise in the parotid gland, paranasal sinuses, orbital cavity, lung and stomach [93].

Many studies have reported an increased risk of gastric cancer in CVID patients. The high incidence of gastric malignancies in CVID patients may be related to high rates of *H. pylori* colonization and alterations in the p53 tumour suppressor gene [97]. The gastrointestinal defects associated with CVID, such as the decreased gastric IgA production, may result in enhanced *H. pylori* colonization and gastric inflammation, thus promoting carcinogenesis [98]. *H. pylori* causes chronic gastritis by stimulating the release of pro-inflammatory cytokines and favouring achlorhydria; besides, certain strains produce virulence factors with oncogenic effects on the gastric epithelium. *H. pylori* induces the upregulation of oncogenes and the silencing of tumour suppressor genes, triggering a stepwise cascade of events ranging from intestinal metaplasia to dysplasia to neoplasia, the so-called Correa’s cascade [99].

CVID patients can also be affected by extranodal marginal zone NHL arising in mucosal sites, named MALT lymphomas or “maltomas”. In CVID patients, 10 cases of extranodal marginal zone lymphoma have been reported [100], but many more cases are probably clinically hidden. Extranodal marginal zone lymphomas are low-grade B cell lymphomas that occur in organs with lymphoid infiltration due to infectious or autoimmune stimulation. A relationship between *H. pylori* infection and extranodal marginal zone lymphoma with gastric location is possible, given that *H. pylori* infection is present in more than 90% of the patients with this type of lymphoma [101].

### 4.3. X-Linked Agammaglobulinemia (XLA)

XLA is a rare primary immunodeficiency disorder with an estimated prevalence of ~1 in 200,000 [102]. First described by the paediatrician Ogdon Bruton in 1952, XLA is caused by germline mutations in the Bruton tyrosine kinase (BTK) gene [103] that result in delayed or blocked development of mature B lymphocytes, absent or very low serum immunoglobulin levels and failure of specific antibody production [104]. BTK plays a crucial role in B cell development as it is required for transmitting signals from the pre-B cell receptor that forms after successful immunoglobulin heavy chain rearrangement [104]. It also has a role in mast cell activation through the high-affinity IgE receptor [104]. Therefore, patients treated with intravenous immunoglobulin replacement therapy present a significant prognostic improvement [105]. Clinically, patients with XLA have recurrent respiratory infections with subsequent risk of developing serious complications, in particular, bronchiectasis. The development of bronchiectasis is a leading cause of mortality and morbidity for these patients, diminishing their quality of life [106].

In an Italian cohort study, malignancies were documented in a minority of cases (3.7%), with four XLA patients who developed gastrointestinal tract’s tumours during follow-up: one gastric adenocarcinoma, one liver carcinoma and two colon adenocarcinomas [107]. From other studies, the rates of malignancy in XLA patients are reported to be between 1.5 and 6%, with patients most likely to develop gastric cancer and colorectal cancer [108]. A role for chronic infection and chronic inflammation has been suggested, similar to cancer’s pathogenesis in inflammatory bowel disease and atrophic gastritis [109,110]. Multiple cases of predominantly left-sided colorectal neoplasia might be a phenotypic expression of XLA, and for patients with such disease, surveillance colonoscopy starting at a young age might be justified.

Finally, BTK has been discovered to serve a critical role in the survival and infiltration of B cell lymphoma [111]. Recently, it was reported that BTK inhibitors exerted potential beneficial effects against numerous types of solid tumour, including glioblastoma multiforme and breast cancer; however, whether BTK is crucial for the progression of bladder cancer remains unclear [111].

### 4.4. Wiskott–Aldrich Syndrome (WAS)

WAS is a rare X-linked primary immunodeficiency characterized by thrombocytopenia, eczema, recurrent infection and an increased incidence of autoimmunity and malignancy. WAS demonstrates an estimated incidence of 1 in every 100,000 live births. WAS is caused by mutations in the WAS gene (located on the short arm of the X chromosome, at Xp11.22-23), encoding Wiskott–Aldrich syndrome protein, which is expressed only in haematopoietic cells and is involved in cellular signalling and immunological synapse formation [112]. The severity of immunodeficiency depends largely on the type of mutation and the resulting protein expression. The different mutations alter the protein’s configuration in several ways, leading to phenotypic variability in disease manifestations, ranging from a severe phenotype (classic WAS) to milder ones such as X-linked thrombocytopenia and X-linked neutropenia [113].

Thrombocytopenia is the most common finding present at the time of diagnosis. WAS patients may present petechial and prolonged bleeding from the umbilical stump or after circumcision, in the first days of life. During the first year of life, in approximately one-half of WAS patients, there is eczema of varying severity that resembles classical atopic dermatitis. Multiple recurrent infections due to encapsulated organisms (such as *Streptococcus pneumoniae, Neisseria meningitidis* and *Haemophilus influenzae*) include acute otitis media, rhinosinusitis, pneumonia, meningitis, sepsis and colitis. This immunodeficiency also predisposes patients to opportunistic infections by *Pneumocystis jirovecii* and *Molluscum contagiosum*, as well as systemic varicella and cytomegalovirus infection. Diseases reported to be associated with autoimmune diseases include haemolytic anaemia, neutropenia, vasculitis involving both small and large vessels, inflammatory bowel disease and renal diseases.

The possibility of cancer occurrence is a significant concern for WAS patients. Malignancies can occur during childhood but are most frequently present in adolescent and young adult males with the classic WAS phenotype. In retrospective studies of patients with severe clinical presentation, the prevalence of malignancy has been reported to be as high as 13–22%, with an average age of onset of 9.5 years [114]. Reduced CTL cytotoxicity may be the main reason for defective immunity against infection and B cell malignancy in WAS patients [115]. In WAS patients, another important cell defect is related to impaired NK cell crosstalk. NK cells require F-actin accumulation to establish polarized contact with target cells and provide a structural synapse to propagate activation signals [116]. These aberrations can result in defective elimination of altered cells (e.g., virally infected) and defective immunity towards cancer cells, which may facilitate neoplasms’ origination and progression. In WAS, lymphomas, lymphoblastic leukaemia, myelodysplasia, myeloproliferative disorders and other non-lymphoreticular malignancies (e.g., seminoma, testicular carcinoma, glioma, neuroma and Kaposi sarcoma) have been described. WAS has been reported to have correlations with EBV-associated LPDs [62]. Lymphomas, predominantly of the NH type and often of the EBV-induced type, are the most frequently diagnosed form of neoplasm and present in extranodal sites. In WAS patients, cytotoxic cell lines show reduced lytic activity against lymphoma cells. Disorganized immunological synapses and incomplete polarization of perforin-containing lytic granules towards tumoral targets are the mechanisms contributing to the development of haematological malignancies in WAS patients.

### 4.5. Chromosome 22q11.2 Deletion Syndrome

Chromosome 22q11.2 deletion syndrome (22q11.2DS) is a multisystem disorder that occurs in approximately 1:4000 births. This disorder includes a variety of phenotypical syndromes with a common genetic deletion; among these syndromes are DiGeorge syndrome and velo-cardio-facial syndrome [117]. Patients with 22q11.2DS have different clinical findings: cardiac anomalies (tetralogy of Fallot, interrupted aortic arch, ventricular septal defect and truncus arteriosus), endocrine anomalies (hypocalcaemia and growth hormone deficiency), palatal anomalies (cleft palate, submucosa cleft palate, velopharyngeal insufficiency and bifid uvula), renal anomalies (absent/dysplastic, obstruction and reflux), ophthalmologic anomalies, neurological anomalies, skeletal anomalies (cervical spine anomalies, vertebral anomalies and lower extremity anomalies), speech delay, developmental delay in infancy and in childhood and behavioural/psychiatric problems [118]. Patients also show variable immune deficiency that is related to the size of the deletion in chromosome 22q11.2. Since they have a hypoplastic thymus, low T cells levels are found; this condition is the most commonly described immunologic feature of 22q11.2DS. People with this syndrome could also have a secondary humoral immune deficiency (compromised differentiation of B cells into the switched-memory compartment and low levels of immunoglobulin) because compromised T cells cannot help B cells in their development [119].

T cell deficits predispose patients to infections, with possible involvement of carcinogenic viruses such as EBV or HPV, and possibly interfere with tumour surveillance. The most frequent malignancies are NHL, HL and acute leukaemia [117].

### 4.6. Ataxia Telangiectasia (A–T)

A–T is a rare autosomal recessive disorder due to biallelic inactivation of the A–T mutated (ATM) serine–threonine protein kinase. This protein is involved in many cellular functions, including activation of cell cycle checkpoints, apoptosis, response to double-strand DNA breaks, oxidative stress and other genotoxic stresses [120]. A–T is characterized by cerebellar degeneration, telangiectasia, immunodeficiency, increased cancer risk and sensitivity to ionizing irradiation. Its prevalence is estimated to be between 1 in 40,000 and 1 in 100,000 live births [121]. Immunological manifestations include low levels of immunoglobulin (IgG, IgG subclasses, IgA and IgM) and lymphopenia. These immunologic alterations lead to an increased risk of infections (particularly sinopulmonary infections) and of autoimmune/chronic inflammatory diseases [121].

Patients with A–T have an increased risk of cancers: lymphoid tumours and a variety of solid tumours, including breast, liver, gastric and oesophageal carcinomas. The high incidence of malignancies in A–T patients is caused by defective cell cycle checkpoint activation, reduced capacity for repair of double-strand (ds)DNA breaks and abnormal apoptosis. Different studies show that lymphomas (HL, B cell NHL) and lymphoblastic leukaemia occur at a higher rate and at an earlier age (median age at diagnosis 10.6–12.6 years) than other malignancies (breast, gastric, thyroid and liver carcinomas and gliomas: median age at diagnosis 31.4 years) [122]. A meta-analysis has assessed the relationship between specific polymorphisms of ATM and the type of cancer: the ATM rs664143 polymorphism is associated with lung cancer risk, and the ATM rs664677 polymorphism is associated with a high risk of breast cancer [123].

### 4.7. WHIM Syndrome

WHIM syndrome is an extremely rare autosomal dominant primary immunodeficiency that results from heterozygous gain-of-function mutations in the chemokine receptor CXCR4. Its incidence is unknown, but it has been estimated to be less than 1:4,000,000 births [124]. The acronym WHIM stands for warts, hypogammaglobulinemia, infections, and myelokathexis. CXCR4 is involved in the retention of neutrophils and other leukocytes in the bone marrow. Gain-of-function mutations of this chemokine receptor exaggerate this process (myelokathexis), resulting in severe peripheral neutropenia and increased susceptibility to infections (e.g., oto-sinopulmonary infections, dental infections, recurrent cellulitis, abscesses or deep tissue infections). Because white blood cells are not released into the bloodstream, patients with WHIM syndrome also have low levels of B lymphocytes; consequently, affected individuals have hypogammaglobulinemia.

Patients with this IEI are particularly susceptible to HPV, which causes warts, and mucosal, oral and genital warts are associated with an increased risk of squamous carcinoma [57]. A study that analysed data collected from an international cohort of 18 patients with CXCR4 mutations showed an incidence of skin warts in 61% of patients and an incidence of HPV-related malignancies in 16% of patients [125]. It would seem that CXCR4 gain-of-function mutations lead to HPV keratinocyte transformation and confer transforming capacity to HPV18-immortalized keratinocytes. In keratinocytes immortalized by high-risk HPV16 or HPV18, CXCR4 and its ligand (CXCL12) are abnormally expressed. In WHIM syndrome, gain-of-function mutations in the chemokine receptor CXCR4 may provide conditions supportive of oncogenic transformation [126]. Patients with WHIM syndrome have also an increased risk of lymphoproliferative diseases/haematological cancers, particularly lymphoma [127]. These haematological malignancies could be related to viral infections: EBV-driven lymphomas presumably develop in an analogous manner to HPV-driven squamous cell carcinoma due to a failure of the immune system to control this infection. The major types of B cell malignancy linked to EBV infection are Burkitt lymphoma, HL and diffuse large B cell lymphoma [128]. Other haematological disorders can arise, such as acute myeloid leukaemia, Waldenstrom macroglobulinemia and multiple myeloma, with CXCR4 and CXCL12 axis involved in tumour progression [129].

## 5. Treatment of Cancer in IEI Patients

The prognosis for patients with IEI and cancer is generally worse than the prognosis of patients with the same malignancies in the general population. Difference in outcome mainly relies on an increased risk of infection, organ damage related to systemic cytotoxic therapy and the onset of secondary (therapy related) neoplasms [22]. Chemotherapy regimens, generally, do not differ from those used for immunocompetent patients, except for an individual modulation of the chemotherapeutic dosage mostly in DNA repair defect and for the execution of an aggressive anti-infective prophylaxis [130]. Very few randomized clinical trials have been performed on IEI patients, but small series and case reports show that response to treatment and prognosis are inferior in IEI patients compared to non-IEI patients due to the inability of patients with IEI to tolerate standard chemotherapy and their susceptibility to life-threatening infections [122]. Short chemotherapy protocols are preferred, and infection control is critical, with a particular concern for prophylaxis against *P. jirovecii* pneumonia [34,131].

When malignancy occurs in IEI patients, additional tests are needed to assess the degree of immune impairment based on the underlying genetic defect. The tests should include evaluation of radiosensitivity, so that appropriate treatment regimens can be formulated. In particular, radiation-based therapies should be avoided in patients with known genetic defects that predispose to radiosensitivity [132].

Myeloablative stem cell transplantation remains the best curative option in patients with IEI who survive lymphoproliferative diseases. The recent introduction of stem cell transplantation with reduced-intensity conditioning associated with rituximab (anti-CD20 monoclonal antibody) for initial treatment of PTLDs in combination with posttransplant EBV suppression proved to be successful [133,134,135]. 

Among the various innovative therapies that have been proposed to treat malignancy in IEI, immunotherapy seems promising, as it could be used to create personalized, chemotherapy-free treatments for lymphoma. Potential immunotherapy options include monoclonal antibody-based immunotherapy (e.g., rituximab, obinutuzumab, andepratuzumab), conjugated antibodies (e.g., brentuximab vedotin), bi-specific T cell engager (BiTE) antibodies (blinatumomab), anti-PD1 antibodies (pembrolizumab and nivolumab), anti-PDL-1 antibodies (atezolizumab and durvalumab) and anti-CTLA4 checkpoint inhibitors (e.g., ipilimumab) [136]. Complete remission with rituximab and brentuximab vedotin was reported in an adult female patient with CVID-associated classic HL, while two other cases of paediatric CVID-associated HL succumbed to severe infection related to chemotherapy [136]. DCs have long been a focus of cancer immunotherapy due to their role in inducing protective adaptive immunity, but cancer vaccines have shown limited efficacy in the past [137,138]. With the advent of immune checkpoint blockade and the ability to identify patient-specific neoantigens, new vaccines and combinatorial therapies are being evaluated in the clinic. Understanding how to augment the function of DCs could offer new approaches to enhance immunotherapy, in addition to improving cytotoxic and targeted therapies that are partially dependent upon a robust immune response for their efficacy [137,138].

Finally, the efficacy of adoptive T cell therapies such as chimeric antigen receptor (CAR) T cells is still uncertain [139]. The role of these targeted therapies is still under discussion, as they could cause an increased risk for tumorigenesis as a result of immune manipulation in the context of an underlying immune deficiency or immune dysregulation [132].

## 6. Conclusions

Immunologists and oncologists should interact to monitor and promptly diagnose the potential development of cancer in known IEI patients, as well as an underlying IEI in patients with newly diagnosed cancers with suggestive medical history or high rate of therapy-related toxicity [139]. As many of both IEI and cancer predisposition genes are still unexplored, the intersection of the relevant lists may represent a significant opportunity for a potential genic and molecular targeted approach. The correct management of these conditions (tumours and cancer predisposition) in IEI patients is represented by tailored approaches. Further molecular biology studies are needed to understand the etiopathogenesis and the molecular pathways of these tumours, with the objective of early recognition of cancer in IEI, optimization of existing therapies and development of new targeted therapies. The creation of an international registry of IEI cases with detailed information on the occurrence of cancer is fundamental to optimizing the diagnostic process and to evaluating the outcomes of new therapeutic options, with the aim of improving prognosis and reducing comorbidities.

## Figures and Tables

**Table 1:** Abbreviations used in this manuscript.

Abbreviation	Full Name
A–T	Ataxia–telangiectasia
ALPS	Autoimmune lymphoproliferative syndrome
ATM	Ataxia–telangiectasia mutated
BLPDs	B cell lymphoproliferative disorders
22q11.2DS	Chromosome 22q11.2 deletion syndrome
CAR	Chimeric antigen receptor
CID	Combined immunodeficiency
CSR	Class switch recombination
CTLs	Cytotoxic T lymphocytes
CVID	Common variable immunodeficiency
DCs	Dendritic cells
DGS	DiGeorge Syndrome
EBNA-2	Epstein–Barr virus nuclear antigen 2
EBV	Epstein–Barr virus
HL	Hodgkin’s lymphoma
HHV	Human herpes virus
HPV	Human papilloma virus
ICR	Immunodeficiency Cancer Registry
IEI	Inborn Errors of Immunity
IFN	Interferon
IL	Interleukin
ITK	Interleukin-2-inducible T-cell kinase
LPDs	Lymphoproliferative disorders
MALT	Mucosa-associated lymphoid tissue
NHL	Non-Hodgkin’s lymphoma
PTLDs	Posttransplant lymphoproliferative disorders
SCID	Severe combined immunodeficiency
SMH	Somatic hypermutation
TCR	T cell receptor
TLR	Toll-like receptor
TNF	Tumour necrosis factor
WAS	Wiskott–Aldrich syndrome
WHIM	Warts, hypogammaglobulinemia, infections, and myelokathexis
XLA	X-Linked Agammaglobulinemia
XLP	X-linked lymphoproliferative syndrome
XMEN	X-linked immunodeficiency with magnesium defect, Epstein-Barr virus infection, and neoplasia

**Table 2:** Malignancy patterns of various primary immunodeficiency subtypes.

Disease	Disease Frequency	Over-Represented Cancers
***Selective IgA deficiency***	1:600	Gastric cancers Lymphoma
***CVID***	1:25–50.000	Lymphoma (more frequently NHL) Gastric cancers Thymic cancers Breast cancers Bladder cancers Cervical cancers
***X-linked agammaglobulinemia***	1:200.000	Gastric cancers Colorectal cancers
***Wiskott-Aldrich syndrome***	1:100.000	Lymphoma Lymphoblastic leukaemia Myelodysplasia-myeloproliferative disorders
***Chromosome 22q11.2 deletion syndrome***	1:4.000	Lymphoma Acute leukaemia
***Ataxia telangiectasia***	1:40.000–100.000	Lymphoma Lymphoblastic leukaemia Breast cancers Liver cancers Gastric cancers Oesophageal cancers Glioma
***WHIM syndrome***	1:4.000.000	Lymphoma Genital and squamous carcinoma Acute myeloid leukaemia
